# Supplementary material for: DeepSelectNet: deep neural network based selective sequencing for oxford nanopore sequencing
Source: BMC Bioinformatics. 2023 Jan 28;24:31. doi: 10.1186/s12859-023-05151-0 (PMC9883605; doi:10.1186/s12859-023-05151-0)
Supplement: Supplementary file 1 — Additional file 1. Supplementary Information. [file 12859_2023_5151_MOESM1_ESM.pdf]

Supplementary Materials - DeepSelectNet: Deep Neural Network Based Selective Sequencing for Oxford Nanopore Sequencing

Anjana Senanayake[1], Hasindu Gamaarachchi[2,3], Damayanthi Herath[1] and Roshan Ragel[1]

1 Department of Computer Engineering, University of Peradeniya, Sri Lanka.
2 Kinghorn Centre for Clinical Genomics, Garvan Institute of Medical Research, Australia.
3 School of Computer Science and Engineering, University of New South Wales, Australia.

List of Tables

S1 Test accuracy comparison of SquiggleNet vs DeepSelectNet across five dataset combinations 1
S2 Test accuracy comparison of DeepSelectNet against existing methods across five dataset combinations 1
S3 Prediction runtime comparison of DeepSelectNet against other methods across five Dataset combinations 1
S4 DeepSelectNet's overall performance across five dataset combinations 2
S5 DeepSelectNet's performance across different read lengths in the Cov&Zymo dataset 2
S6 DeepSelectNet's performance across different number of read samples in Cov&Zymo dataset 2
S7 Impact of Median Absolute Deviations(MAD) on DeepSelectNet's accuracy 3
S8 Training Accuracy of DeepSelectNet's across five cross folds 3
S9 Performance of DeepSelectNet's across dataset combinations including Human 3
S10 Accuracies of DeepSelectNet's before and after introducing segment sampling 4
S11 DeepSelectNet performance for artificially generated signals for Yeast & Chlamydomonas 4
S12 Genome Coverage of the individual species used in dataset combinations 4
S13 Genome Coverage of Covid & Zymo for different read lengths 5
S14 Genome Coverage of Covid & Zymo for different number of reads 5
S15 DeepSelectNet's overall performance for Zymo intra species Bacillus subtilis and Saccharomyces cerevisiae 5

List of Figures

S1 Adapter segment in the first 1000-1500 signal samples in reads 6
S2 Outliers in sequence signal 6

S3 Box plot of normalized raw signals for empirically deriving the optimal MAD threshold values........ 7
S4 Accuracy, Precision, Recall and F1 Score comparison of SquiggleNet vs DeepSelectNet..... 8
S5 Accuracies of different baseline methods across 5 datasets (for complete read)............... 9
S6 Accuracies for all methods across 5 datasets (for 300 bases).. 10
S7 Accuracy, Precision, Recall, and F1 Score comparison of all existing methods against DeepSelectNet........ 11
S8 Inference runtime comparison of DeepSelectNet against other methods across five dataset combinations)........ 12

Supplementary Notes

Supplementary Note 1 - Instructions to run the tools........ 13

Table S1. Test accuracy comparison of SquiggleNet vs DeepSelectNet across five dataset combinations

Dataset	Squigglenet	DeepSelecNet
**Covid & Zymo**	79.69%	91.28%
**Zymo & Chlamydomonas**	94.81%	97.31%
**Covid & Yeast**	79.83%	90.9%
**Covid & Chlamydomonas**	96.82%	98.65%
**Yeast & Chlamydomonas**	92.69%	96.93%

Table S2. Test accuracy comparison of DeepSelectNet against existing methods across five dataset combinations

Datasets	Baseline	Guppy_hac+ Minimap2	SquiggleNet	DeepSelectNet	Guppy_fast+ Minimap2
**Covid & Zymo**	95.76%	93.53%	79.69%	91.28%	90.95%
**Zymo & Chlamydomonas**	96.58%	93.03%	94.81%	97.31%	91.46%
**Covid & Yeast**	91.4%	87.53%	79.83%	90.9%	86.79%
**Covid & Chlamydomonas**	86.94%	80.96%	96.82%	98.65%	77.83%
**Yeast & Chlamydomonas**	87.94%	80.62%	92.69%	96.93%	78.49%

Table S3. Prediction runtime comparison of DeepSelectNet against other methods across five dataset combinations

Dataset	SquiggleNet	DeepSelectNet	Guppy_hac+ Minimap2	Guppy_fast+Minimap2
**Covid & Chlamydomonas**	0.00355 ms	0.003 ms	0.0105 ms	0.000375 ms
**Covid & Yeast**	0.004025 ms	0.001975 ms	0.004925 ms	0.0002 ms
**Covid & Zymo**	0.001325 ms	0.00175 ms	0.00345 ms	0.000275 ms
**Yeast & Chlamydomonas**	0.00395 ms	0.00345 ms	0.014725 ms	0.000375 ms
**Zymo & Chlamydomonas**	0.00385 ms	0.003175 ms	0.013225 ms	0.00045 ms

Table S4. DeepSelectNet's overall performance across five dataset combinations

Dataset	Accuracy	Precision	Recall	F1 Score
**Covid & Zymo**	91.28%	91%	91.62%	91.31%
**Zymo & Chlamydomonas**	97.31%	97.9%	96.69%	97.29%
**Covid & Yeast**	90.9%	89.67%	92.45%	91.04%
**Covid & Chlamydomonas**	98.65%	97.93%	99.39%	98.65%
**Yeast & Chlamydomonas**	96.93%	96.78%	97.08%	96.93%

Table S5. DeepSelectNet's performance across different read lengths in the Cov&Zymo dataset

Read Length	Accuracy
1000	76.71%
1500	82.3%
2000	87.52%
2500	90.06%
3000	91.14%
3500	93.15%
4000	94.92%
4500	95.07%

Table S6. DeepSelectNet's performance across different number of read samples in Cov&Zymo dataset

Number of Reads	Accuracy
2500	80.52%
5000	83.53%
7500	87.09%
10000	87.3%
12500	88.88%
15000	88.86%
17500	90.85%
20000	91.59%

Table S7. Impact of Median Absolute Deviations(MAD) on DeepSelectNet's accuracy

Dataset	MAD = 3	MAD = 5	MAD = 10
**Covid & Zymo**	91.28%	90.09%	91.68%
**Zymo & Chlamydomonas**	97.31%	97.22%	97.62%
**Covid & Yeast**	90.9%	91.58%	91.5%
**Covid & Chlamydomonas**	98.65%	99.06%	98.92%
**Yeast & Chlamydomonas**	96.93%	96.97%	96.87%

Table S8. Training Accuracy of DeepSelectNet's across five cross folds

Dataset	Fold 1	Fold 2	Fold 3	Fold 4	Fold 5
**Covid & Zymo**	92.31%	92.19%	92.29%	92.34%	92.48%
**Zymo & Chlamydomonas**	97.3%	97.35%	97.05%	97.26%	97.11%
**Covid & Yeast**	91.62%	91.81%	91.99%	91.32%	92.2%
**Covid & Chlamydomonas**	98.82%	99.1%	99.11%	99.09%	99.01%
**Yeast & Chlamydomonas**	96.73%	96.81%	96.8%	96.97%	96.7%

Table S9. Performance of DeepSelectNet's across dataset combinations including Human

Dataset	Accuracy	Precision	Recall	F1 Score
**Human& Covid**	96.27%	96.19%	96.35%	96.27%
**Human & Zymo**	92.26%	93.24%	91.13%	92.17%
**Human & Yeast**	81.82%	83.94%	78.68%	81.23%
**Human & Chlamydomonas**	91.19%	89.24%	93.68%	91.41%

Table S10. Accuracies of DeepSelectNet's before and after introducing segment sampling

	DeepSelectNet Accuracy	
**Dataset**	**Before**	**After**
**Covid & Zymo**	84.19%	91.28%
**Zymo & Chlamydomonas**	95.54%	97.31%
**Covid & Yeast**	82.88%	90.90%
**Covid & Chlamydomonas**	97.68%	98.65%
**Yeast & Chlamydomonas**	94.48%	96.93%

Table S11. DeepSelectNet performance for artificially generated signals for Yeast & Chlamydomonas

Species	Accuracy
Yeast	97.39%
Chlamydomonas	96.82%
Yeast & Chlamydomonas	97.10%

Table S12. Genome Coverage of the individual species used in dataset combinations

Dataset	Genome Coverage
Chlamydomonas	0.22
Covid	800

Yeast	0.67
Zymo	0.4

Table S13. Genome Coverage of Covid & Zymo for different read lengths

	Genome Coverage	
**Read Length**	**Covid**	**Zymo**
1000	266.6666667	0.1290322581
1500	400	0.1935483871
2000	533.3333333	0.2580645161
2500	666.6666667	0.3225806452
3000	800	0.3870967742
3500	933.3333333	0.4516129032
4000	1066.666667	0.5161290323
4500	1200	0.5806451613

Table S14. Genome Coverage of Covid & Zymo for different number of reads

	Genome Coverage	
**Number of Reads**	**Covid**	**Zymo**
2500	666.6666667	0.3225806452
5000	1333.333333	0.6451612903
7500	2000	0.9677419355
10000	2666.666667	1.290322581
12500	3333.333333	1.612903226
15000	4000	1.935483871
17500	4666.666667	2.258064516
20000	5333.333333	2.580645161

Table S15. DeepSelectNet's overall performance for Zymo intra species Bacillus subtilis and Saccharomyces cerevisiae

Dataset	Accuracy	Precision	Recall	F1 Score
**Bacillus & Saccharomyces**	82.51%	84.32%	79.87%	82.03%

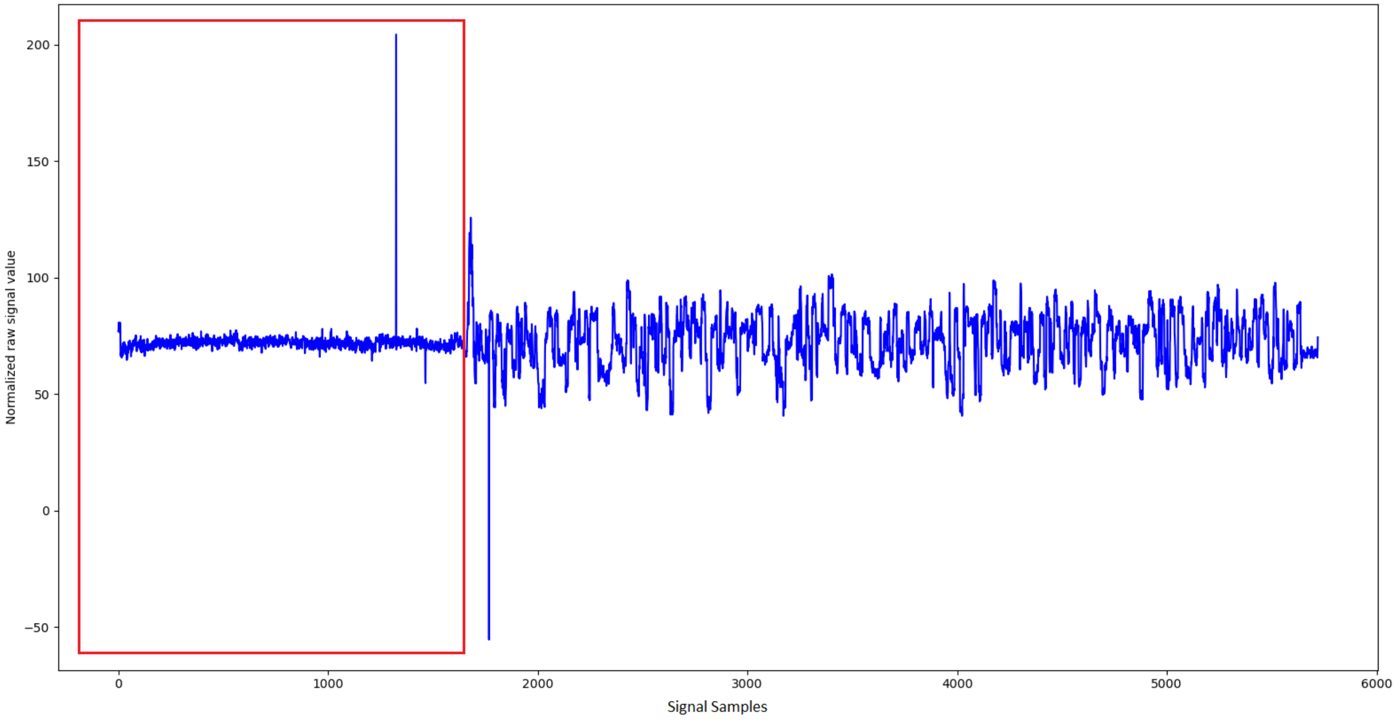

Figure S1. Adapter segment in the first 1000-1500 signal samples in reads

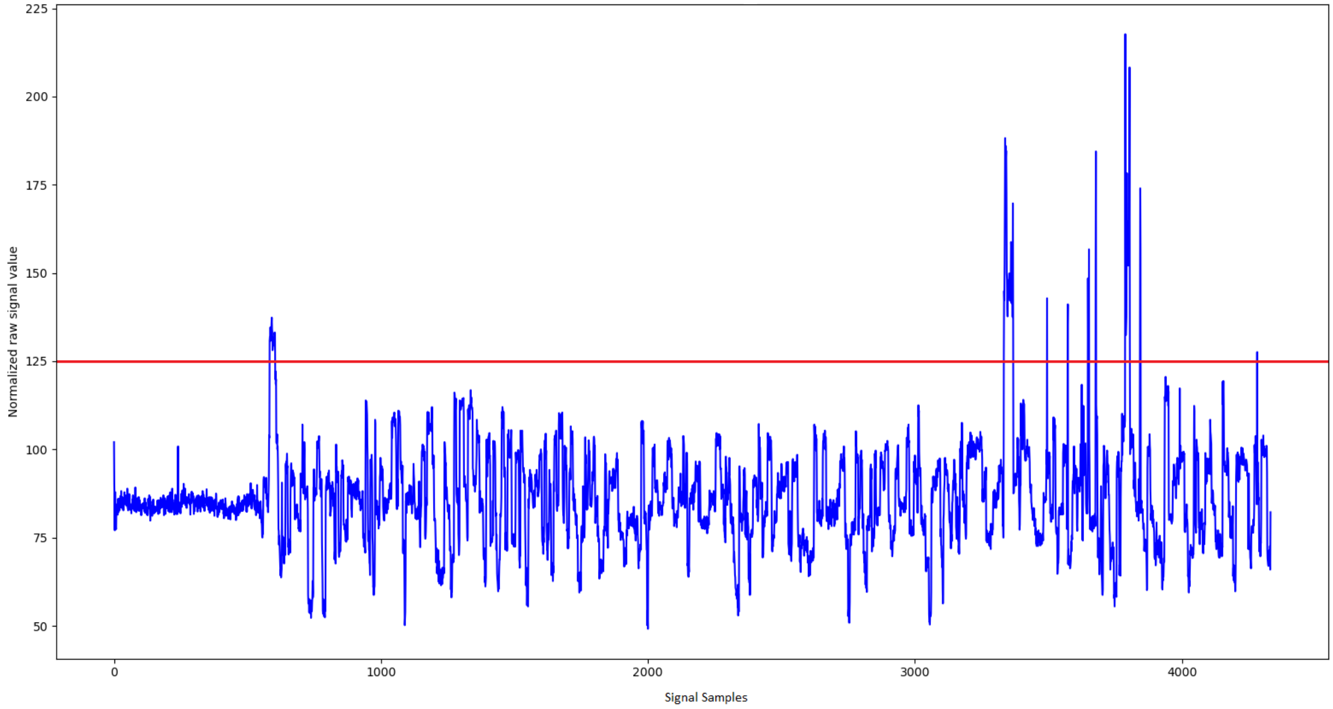

Figure S2. Outliers in sequence signal

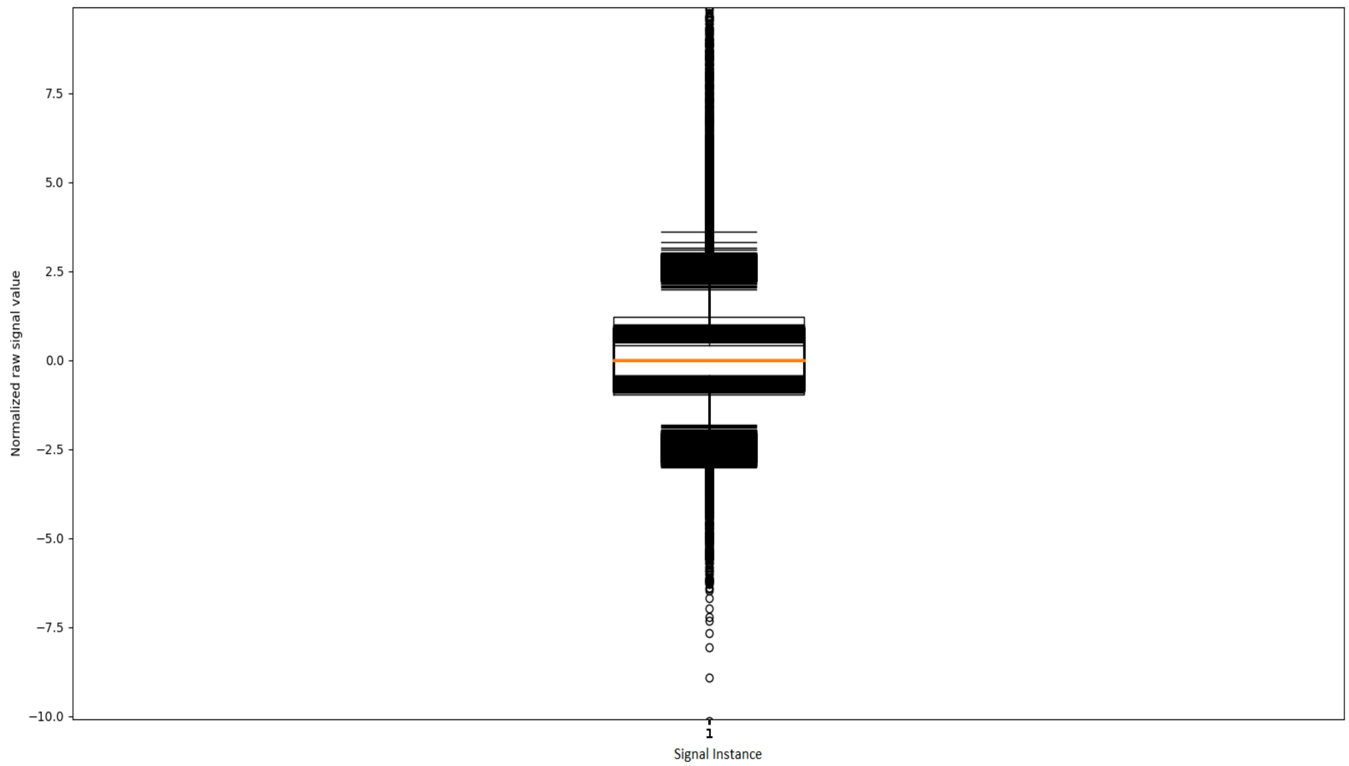

Figure S3. Box plot of normalized raw signals for empirically deriving the optimal MAD threshold values

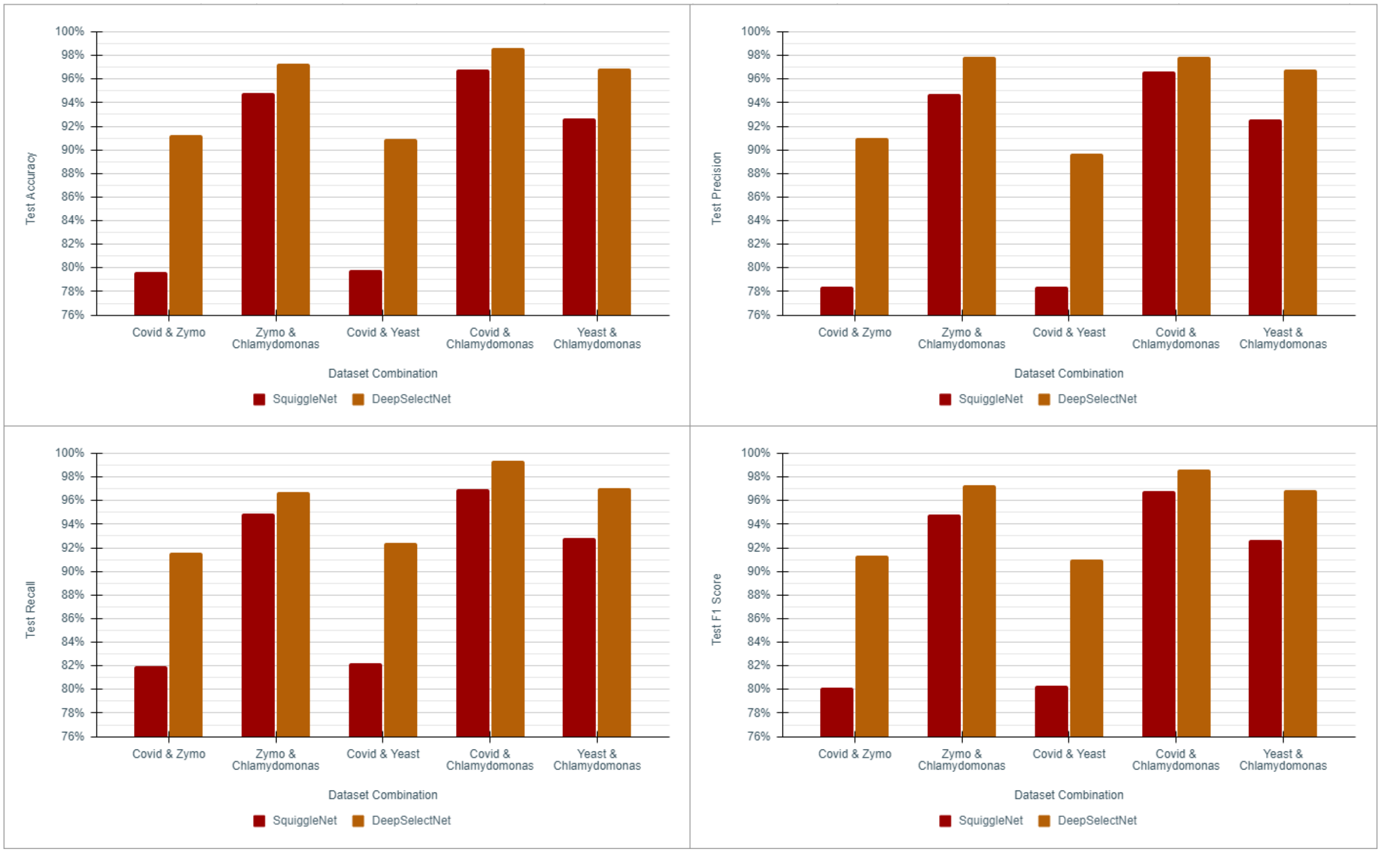

Figure S4. Accuracy, Precision, Recall, and F1 Score comparison of SquiggleNet vs DeepSelectNet

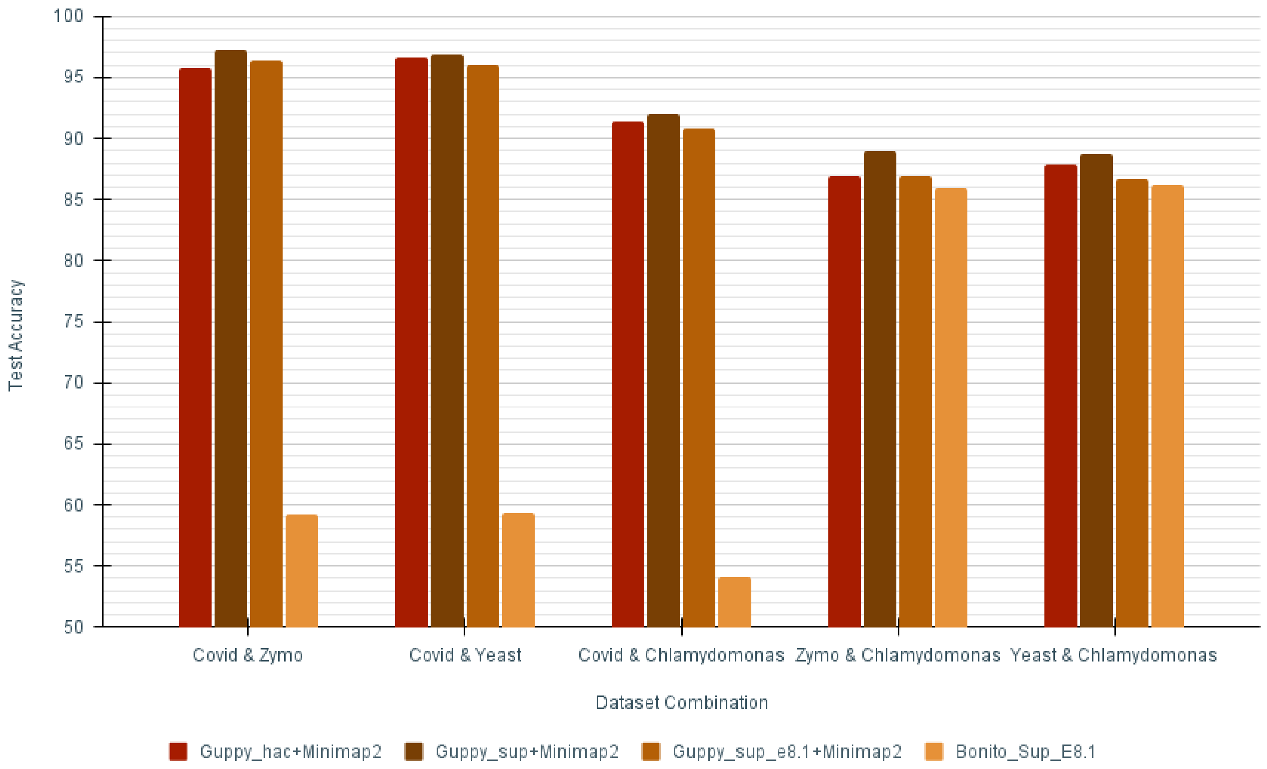

Figure S5. Accuracies of different baseline methods across 5 datasets(for complete read)

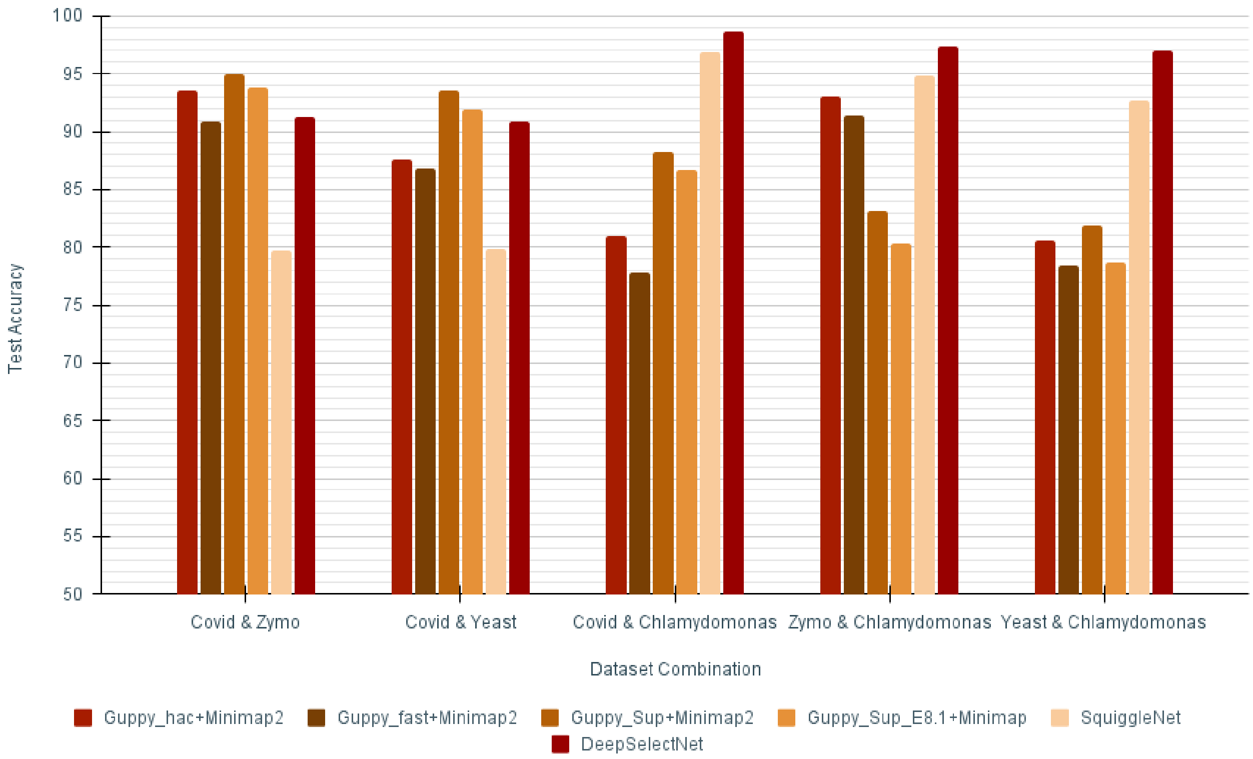

Figure S6. Accuracies for all methods across 5 datasets (for 300 bases)

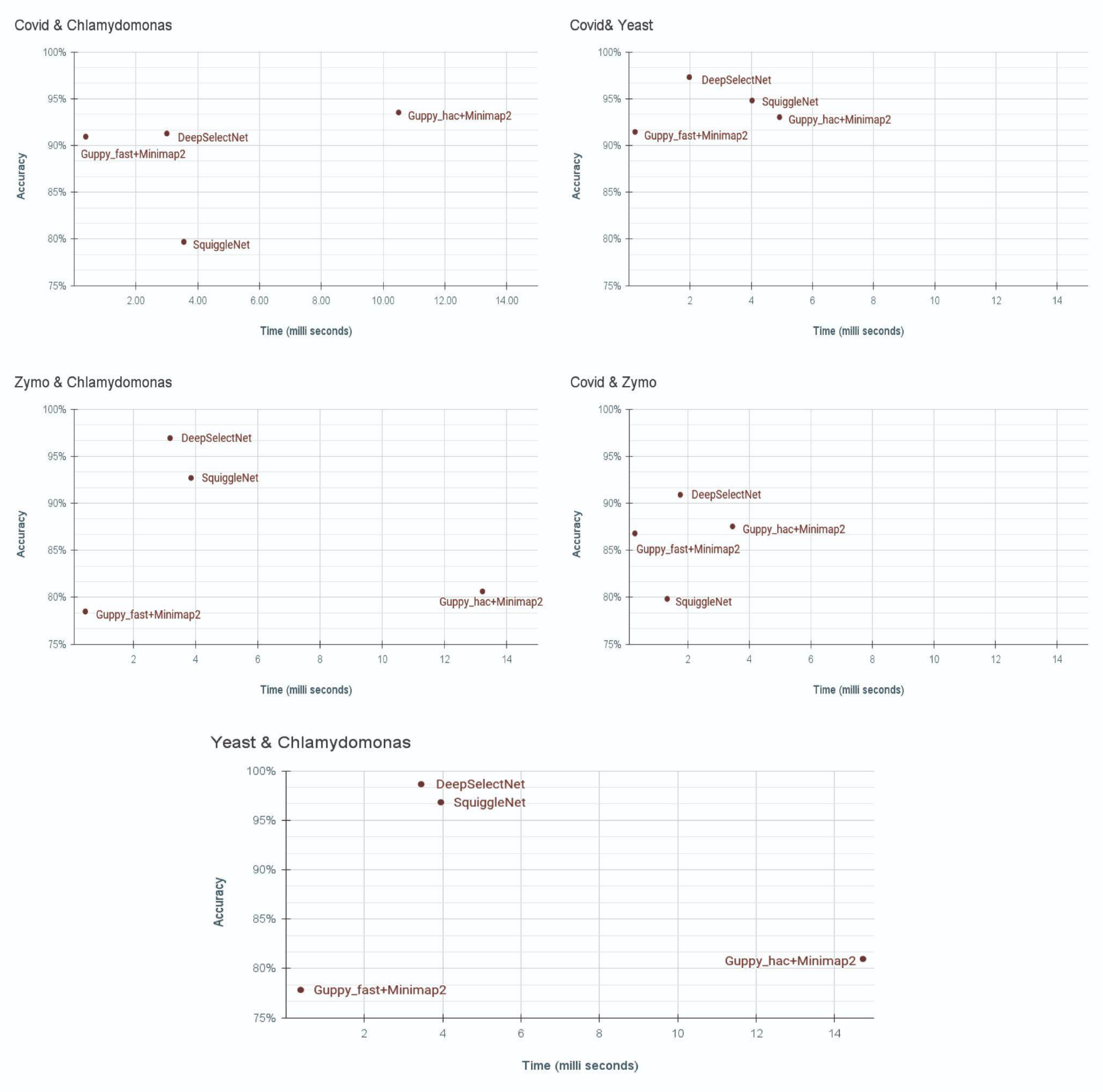

Figure S7. Accuracy, Precision, Recall, and F1 Score comparison of all existing methods against DeepSelectNet

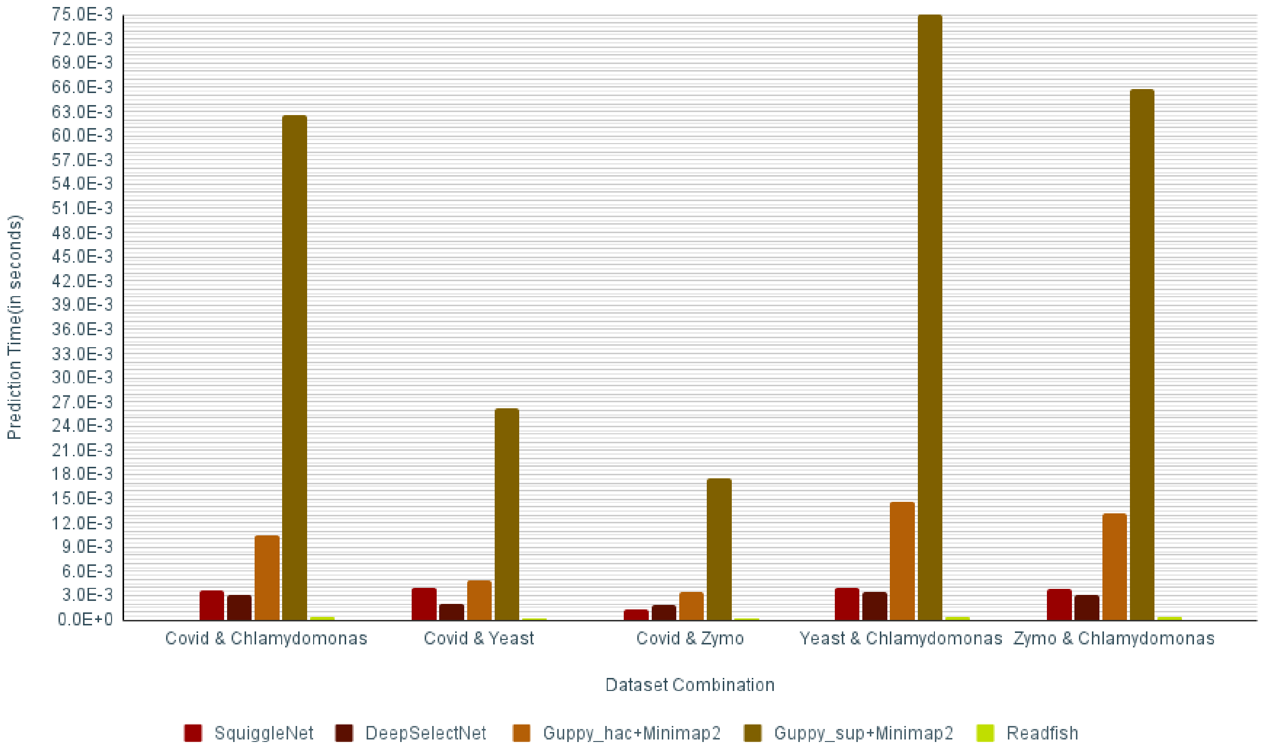

Figure S8. Inference runtime comparison of DeepSelectNet against other methods across five dataset combinations)

Supplementary Note 1 - Instructions to run the tools

Prerequisites

1. Download and install SLOW5 Tools toolkit.

```
#install HDF5 and zlib development libraries
sudo apt-get install libhdf5-dev zlib1g-dev

VERSION=v0.6.0

wget
"https://github.com/hasindu2008/slow5tools/releases/download/$VERSION/slow5too
ls-$VERSION-release.tar.gz" && tar xvf slow5tools-$VERSION-release.tar.gz &&
cd slow5tools-$VERSION/

./configure

make
```

2. Download dataset and extract. Let's call this directory `<DATASET_DIR>`.

```
wget https://doi.org/10.5281/zenodo.7111366
tar xvf DeepSelectNet_curated_datasets.tar <DATASET_DIR>
```

DeepSelectNet

1. Download DeepSelectNet from Github repository.

```
git clone git@github.com:AnjanaSenanayake/DeepSelectNet.git

cd DeepSelectNet
```

2. Set up the environment for DeepSelectNet by following the instructions here.

3. Preprocess datasets with DeepSelectNet preprocessor.

```
python scripts/preprocessor.py -pos_s5 <DATASET_DIR>/COVID/train-covid -neg_s5
<DATASET_DIR>/ZYMO/train-zymo -b 20000 -c 1500 -sco 4 -mad 5 -o train-dump
```

4. Train the model with preprocessed datasets.

```
python scripts/trainer.py -d train-dump -s 0.7 -k 5 -e 200 -o trainedModel
```

5. Testing the model with the best-trained model.

```
python scripts/inference.py -model trainedModel/<best_model> -s5
<DATASET_DIR>/COVID/test-covid -lb 1 -mad 5 -o predicts-covid.txt

python scripts/inference.py -model trainedModel/<best_model> -s5
<DATASET_DIR>/ZYMO/test-zymo -lb 1 -mad 5 -o predicts-zymo.txt
```

SquiggleNet

1. Download SquiggleNet from Github repository.

```
git clone -b slow5-support https://github.com/AnjanaSenanayake/SquiggleNet.git

cd SquiggleNet
```

2. Set up the environment for SquiggleNet by installing the package requirements here.

3. Splitting datasets to preprocess with SquiggleNet.

```
slow5tools view <DATASET_DIR>/COVID/train-covid | grep -v '^[#@]' | awk
'{print $1}' > read-ids-covid.txt

slow5tools view <DATASET_DIR>/COVID/train-zymo.blow5 | grep -v '^[#@]' | awk
'{print $1}' > read-ids-zymo.txt

cat read-ids-covid.txt | head 14000 > train-covid-squigglenet.txt
cat read-ids-covid.txt | tail 6000 > val-covid-squigglenet.txt
cat read-ids-zymo.txt | head 14000 > train-zymo-squigglenet.txt
cat read-ids-zymo.txt | tail 6000 > val-zymo-squigglenet.txt

mkdir train-covid-zymo
cp <DATASET_DIR>/COVID/train-covid.blow5 train-covid-zymo
cp <DATASET_DIR>/ZYMO/train-zymo.blow5 train-covid-zymo
slow5tools merge train-covid-zymo -o train-covid-zymo.blow5
```

```
mkdir test-covid-zymo
cp <DATASET_DIR>/COVID/test-covid.blow5 test-covid-zymo
cp <DATASET_DIR>/ZYMO/test-zymo.blow5 test-covid-zymo
slow5tools merge test-covid-zymo -o test-covid-zymo.blow5
```

4. Preprocess datasets with SquiggleNet preprocessor.

```
python preprocess.py -b 14000 -ft slow5 -gp train-covid-squigglenet.txt -gn
train-zymo-squigglenet.txt -i train-covid-zymo.blow5 -o output

python preprocess.py -b 6000 -ft slow5 -gp val-covid-squigglenet.txt -gn
cal-zymo-squigglenet.txt -i train-covid-zymo.blow5 -o output
```

5. Training datasets with SquiggleNet.

```
python trainer.py -tt output/pos_14000.pt -nt output/neg_14000.pt -tv
output/pos_6000.pt -nv output/neg_6000.pt -o trainedModel.ckpt
```

6. Training datasets with SquiggleNet.

```
python inference.py -m trainedModel.ckpt -ft slow5 -b 1000 -i
test-covid-zymo.blow5 -o predictions
```

Baseline

```
cd DeepSelectNet/support

sh baseline.sh <DATASET_DIR>/COVID/test-covid.blow5
<DATASET_DIR>/ZYMO/test-zymo.blow5 <DATASET_DIR>/COVID/test-covid.fastq
<DATASET_DIR>/ZYMO/test-zymo.fastq  <DATASET_DIR>/COVID/covid-ref.fasta
<DATASET_DIR>/ZYMO/zymo-ref.fasta
```

Guppy_hac+Minimap2

```
cd DeepSelectNet/support

sh baseline.sh <DATASET_DIR>/COVID/test-covid.blow5
<DATASET_DIR>/ZYMO/test-zymo.blow5 <DATASET_DIR>/COVID/test-covid.fastq
<DATASET_DIR>/ZYMO/test-zymo.fastq  <DATASET_DIR>/COVID/covid-ref.fasta
<DATASET_DIR>/ZYMO/zymo-ref.fasta 300
```

Guppy_fast+Minimap2

```
cd DeepSelectNet/support

sh readfish.sh <DATASET_DIR>/COVID/test-covid.fastq
<DATASET_DIR>/ZYMO/test-zymo.fastq <DATASET_DIR>/COVID/covid-ref.fasta
<DATASET_DIR>/ZYMO/zymo-ref.fasta
```
